# Plasma Exosome-Derived SENP1 May Be a Potential Prognostic Predictor for Melanoma

**DOI:** 10.3389/fonc.2021.685009

**Published:** 2021-08-05

**Authors:** Hejuan Hu, Bai Ling, Yuhan Shi, Haohao Wu, Bingying Zhu, Yiling Meng, Guo-Ming Zhang

**Affiliations:** ^1^Suzhou Key Laboratory for Medical Biotechnology, Suzhou Vocational Health College, Suzhou, China; ^2^Department of Pharmacy, The Yancheng Clinical College of Xuzhou Medical University, Yancheng, China; ^3^Department of Pharmacy, The First People’s Hospital of Yancheng City, Yancheng, China; ^4^Department of Pathology, Shanghai Public Health Clinical Center, Fudan University, Shanghai, China; ^5^Department of Oncology, The Yancheng Clinical College of Xuzhou Medical University, Yancheng, China; ^6^Department of Oncology, The First People’s Hospital of Yancheng City, Yancheng, China; ^7^Department of Laboratory Medicine, The Affiliated Shuyang Hospital of Xuzhou Medical University, Shuyang, China; ^8^Department of Laboratory Medicine, Shuyang People’s Hospital, Jiangsu, China

**Keywords:** SENP1, melanoma, exosomes, prognostic predictor, SUMO

## Abstract

**Objective:**

To evaluate plasma exosome-derived SUMO-specific protease (SENP)1 levels and assess their prognostic value in melanoma.

**Patients and Methods:**

We extracted exosomes from the plasma of 126 melanoma patients, and identified them with transmission electron microscopy, nanoparticle tracking analysis and western blotting. The plasma exosome-derived SENP1 levels of melanoma patients and healthy controls were detected with ELISA.

**Results:**

Plasma exosome-derived SENP1 levels in melanoma patients were significantly upregulated than in healthy controls (*P* < 0.001). Plasma exosome-derived SENP1 levels in melanoma patients with tumor size >10 cm, located in the mucosa or viscera, with Clark level IV/V, with lymph node metastasis, and TNM stages IIb–IV were significantly higher than in patients in with tumor size <10 cm, located in the skin, with Clark level I–III, without lymph node metastasis, and TNM stages IIb–IV (all *P* < 0.05). Disease-free survival (DFS) and overall survival (OS) were worse in melanoma patients who had higher plasma exosome-derived SENP1 levels than lower plasma exosome-derived SENP1 levels (both *P* < 0.001). Area under the receiver operating characteristic curve (AUROC) of plasma exosome-derived SENP1 for predicting 3-year DFS of melanoma patients was 0.82 [95% confidence interval (CI): 0.74–0.88], with a sensitivity of 81.2% (95% CI: 69.9–89.6%) and specificity of 75.4% (95% CI: 62.2–85.9%). The AUROC of plasma exosome-derived SENP1 for predicting 3-year OS of melanoma patients was 0.76 (95% CI: 0.67–0.83), with a sensitivity of 95.7% (95% CI: 85.5–99.5%) and specificity of 62.0% (95% CI: 50.4–72.7%).

**Conclusions:**

Melanoma patients with higher plasma exosome-derived SENP1 levels had worse DFS and OS. The plasma exosome-derived SENP1 levels may be a potential prognostic predictor for 3-year DFS and 3-year OS of melanoma.

## Introduction

Melanoma is a malignant tumor caused by abnormal differentiation of melanocytes (1). Melanocytes exist in the neural crest of the embryo and migrate to many parts of the body during fetal development; mainly in the basal epidermis, hair follicles, mucosal surfaces, meninges and the choroidal layers of the eyes (2, 3). Melanoma is most common in the skin, but the rectum, eyes, anus and vulva are also frequent sites. Malignant melanoma of eyelid skin is rare, accounting for only 1% of eyelid skin malignant tumors (4, 5). Melanoma is difficult to detect during onset and has a high degree of malignancy. At present, its incidence is continuing to increase considerably at a rate of 3–8% per year, even as that of many other tumors declines (6). A century ago, melanoma was still a rare cancer, but the average incidence in western caucasian people reached 1 in 50 by the beginning of the 21st century (7).

According to data from Surveillance, Epidemiology, and End Results Program (SEER) in 2007, 1 in 63 Americans had melanoma (8). The incidence of melanoma was 27.5 per 100,000 in caucasian and 1.1 per 100,000 in black people (9). In the global statistics for malignant melanoma in 2012, 232,000 new cases and 55,000 deaths were reported, with a high fatality rate of 23.7% (10). Melanoma is considered a highly aggressive and metastatic disease with a survival rate of <10% for ≥5 years (11). After melanoma metastasizes, the life expectancy of patients generally does not exceed 1 year (12). In addition, approximately one-third of melanoma patients relapse. Although almost all organs can be affected, the most common target sites are the liver, bone and brain (13). Advances in the primary pathogenesis and therapeutic intervention have allowed melanoma patients to receive better treatment, including targeted therapies such as BRAF and MEK inhibitors, new immunotherapies such as anti-CTLA4 or anti-PD1 therapy, radiation therapy, biochemical therapy, and gene therapy (14). These treatments have improved the progression-free survival and overall survival (OS) rates of patients with advanced unresectable melanoma, but melanoma is still a deadly cancer, especially when patients are diagnosed at an advanced stage (15).

The post-translational modifications of proteins include methylation, phosphorylation and threacylation. Small ubiquitin-related modifier (SUMO) modification is one of the post-translational modifications of proteins (16). By covalently modifying several amino acid residues on the substrate protein, it can change its activity, stability and intracellular localization, and participate in the regulation of various cellular processes (17). Once the dynamic balance between SUMO and de-SUMO is broken, it can lead to tumor occurrence. Sumoylation is catalyzed by E1, E2 and E3 enzymes, while desumoylation is performed by SUMO-specific proteases (SENPs) (18). Human desumoylation proteases SENPs, including SENP1, SENP 2, SENP 3, SENP 5, SENP 6 and SENP 7, play a key role in this pathway (19). One is to catalyze the transformation of SUMO precursor into its active form; the other is to cut off the isopeptide bond between SUMO and the target protein to realize desumoylation (20). SENP is involved in the regulation of the cell cycle, cell proliferation, oxidative stress signaling or tumor gene fusion, leading to tumor formation. SENP family members play different roles (21, 22). SENP1 is a nuclear protein, which can catalyze the desumoylation of SUMO1, SUMO2 and SUMO3 modified target proteins (23). In recent years, several studies have reported that SENP1 is highly expressed in prostatic and pancreatic cancer and osteosarcoma, and knockout of SENP1 affects the biological function of these tumors (24, 25).

Exosomes are small membranous vesicles ranging from 40 to 100 nm (26). They can be used as functional mediators in cell interactions leading to cancer metastasis (27). Metastasis is a complex multistep process of cancer cell invasion, vascular survival, adhesion and host organ colonization. Exosomes affect every step of this cascade, and play an important role in cell-to-cell communication, which can be targeted by tumor therapy (28). A large number of studies have found that exosomes contain many important proteins, which can be used for early diagnosis of tumor, prognostic analysis of patients and targeting by tumor therapy (29, 30). In this study, we aimed to investigate plasma exosome-derived SENP1 levels and determine their prognostic value in melanoma patients.

## Materials and Methods

### Patients and Clinical Samples

We collected blood samples from 126 melanoma patients from the First People’s Hospital of Yancheng City, Shanghai Public Health Clinical Center and Shuyang People’s Hospital from December 2015 to January 2020. The inclusion criteria were as follows: (1) first onset; (2) age ≥18 years; (3) diagnosed clinically and pathologically; (4) patients underwent surgical resection; (5) no preoperative hormone therapy, radiotherapy or chemotherapy; and (6) clinical data were complete. The exclusion criteria were as follows: (1) other types of malignant tumor; (2) congenital diseases, heart, lung and other serious organ dysfunction; (3) liver and kidney function were not complete; (4) preoperative hormone therapy, radiation therapy or chemotherapy; (5) clinical data were incomplete; and (6) lost to follow-up.

The baseline clinical data of 126 melanoma patients were collected from medical records including demographic features, tumor size, lymph node metastasis, American Joint Committee on cancer (AJCC) TNM stages, and pathological differentiation. Patients were followed up to January 2021, with a median follow-up duration of 42.5 months (range: 12.0–72.0 months). The survival data were collected from follow-up records, and disease-free survival (DFS) and overall survival (OS) were calculated. DFS was defined as the duration from resection to disease recurrence, disease progression, or death. OS was defined as the time interval from resection to death. The follow-up results of the 120 patients enrolled in this study were obtained through medical records or telephone interviews.

In addition, we collected blood samples from 50 healthy people (median age 66 years, range 50–73 years) in the First People’s Hospital of Yancheng City. All specimens were enrolled after obtaining informed consent from the patients or their family. The study was approved by the Ethics Committees of the First People’s Hospital of Yancheng City [identification nos. HMU (Ethics) 2017-K044].

### Plasma Exosome Isolation

After fasting for more than 12 hours, 5 ml of peripheral venous blood was collected by EDTA-K2 anticoagulant vacuum blood collection method. The plasma samples were centrifuged at 2500 rpm for 10 min. 5-20 ml of human plasma (1 × PBS diluted 5 times), 500 × g, 4°C centrifugation was carried out for 5 min at room temperature. Take the supernatant to a new centrifuge tube and mix it in 2000 × g. Centrifugation at 4°C for 10 min. Take the supernatant to a new centrifuge tube and add it at 10000×g. Further centrifugation at 4°C for 30 min removed large cell vesicles. 45μm filter is used to filter the supernatant to remove the large particles that may be mixed in the operation process. Take the filtrated supernatant to the ultracentrifugation tube and use 1×PBS buffer was filled up, weighed accurately and balanced, then put it on the rotor of the ultracentrifuge, 100,000×g Centrifugation at 4°C for 2 hours. The supernatant was discarded, and translucent sediments were found at the bottom of the tube. Resuspension the sediment in 1 × PBS buffer, and in 100,000×g Centrifugation at 4°C was carried out for 80 min at room temperature.

### Transmission Electron Microscopy (TEM)

TEM was used to observe the morphology of exosomes. The extracted exosomes were removed from the −80°C freezer and thawed. Exosome suspension (20 μl) was added to the copper mesh. The solution was kept at room temperature for 1 min. The liquid was removed from the side with filter paper. Phosphotungstic acid solution (30 μl, pH = 6.8) was added to the copper mesh. The solution was negatively stained at room temperature for 1 min. The filter paper was used to absorb the negative dye solution, and the working voltage was adjusted to 75 kV after 10 min exposure to an incandescent lamp.

### Nanoparticle Tracking Analysis (NTA)

NTA was used to observe the size of exosomes. After thawing, 500 μl exosomes was placed in the Nano Sight NS300 Instrument. We set the parameters to detect the outer body particle size. The experimental data were recorded and analyzed with NTA 3.3 software.

### Western Blotting

Western blotting was used to confirm the exosome purification. Exosomes were isolated and added to sodium dodecyl sulfate (SDS) buffer to obtain total proteins. Total protein was separated with SDS-PAGE and transferred to polyvinylidene difluoride membranes (Millipore, USA). The membranes were incubated in 5% skimmed milk for 1 h and then overnight with primary antibodies against annexin V, TSG101, CD9 and CD63 obtained from Santa Cruz Biotechnology, Inc. (TX, USA). Finally, the secondary antibody was added used to the membranes at room temperature for 1 h.

### ELISA

After shaking and mixing, the sample was diluted with PBS (1:3 dilution). The standard and blank controls were added to the microplate coated with SENP1 antibody. Diluted exosome samples (100 μl) were incubated at 37°C for 1 h, the liquid in the microplate was shaken off, pat dry, added solution A (sodium acetate 13.6g, citric acid 1.6g, 30% hydrogen peroxide 0.3ml, distilled water to 500ml), incubated at 37 °C for 1 h, washed three times, added solution B (EDTA disodium 0.2g, citric acid Dao 0.95g, glycerin 50ml, take 0.15g TMB to dissolve in 3ml DMSO, add distilled water to 500ml), incubated at 37°C for 30 min, washed five times, added 90 μl substrate, incubated at 37°C for 15 min, added 50 μl of termination solution, and measured optical density at 450 nm wavelength immediately.

### Statistical Analysis

SPSS 24.0 software (IBM) was performed for statistical analysis. Data were presented as mean ± standard deviation, median (range), or count (percentage). Chi square test or Wilcoxon’s rank-sum test was performed to analyze the correlation. The differences in DFS and OS between two groups were assessed with log-rank test. Receiver operating characteristic (ROC) curve analysis was used to assess the prognostic value of plasma exosome-derived SENP1 levels in melanoma patients. Differences with *P* < 0.05 were considered statistically significant.

## Results

### Baseline Characteristics

The baseline characteristics of 126 patients with melanoma are shown in **Table 1**, including 68 men (63.97%) and 58 women (46.03%); 49 patients (38.89%) aged <60 years, and 77 (61.11%) were ≥60 years. The tumor size was <10 cm in 113 cases and ≥10 cm in 13. Ninety-eight cases (77.78%) were located in the skin, and 28 (22.22%) in the mucosa or viscera. Sixty cases (47.62%) were Clark level I–III and 66 (52.38%) were Clark level IV/V. There were 69 cases (54.76%) with lymph node metastasis. In addition, 57 (45.24%) and 69 (54.76%) cases were stage 0–IIa and IIb–IV, respectively.

**Table 1:** Baseline characteristics of enrolled melanoma patients.

Characteristic	Melanoma patients (n = 126)
**Gender**	
Male	68 (53.97)
Female	58 (46.03)
**Age (years)**	
<60	49 (38.89)
≥60	77 (61.11)
**Tumor diameter (cm)**	
<10	113 (89.68)
≥10	13 (10.32)
**Tumor location**	
Skin	98 (77.78)
Mucous membrane, viscera	28 (22.22)
**Depth of tumor invasion (Clark level)**	
I-III	60 (47.62)
IV-V	66 (52.38)
**Lymph node metastasis**	
YES	69 (54.76)
NO	57 (45.24)
**Tumor stage**	
0-IIa	57 (45.24)
IIb-IV	69 (54.76)

### Characterization of Exosomes Isolated From Plasma

**Figure 1:**
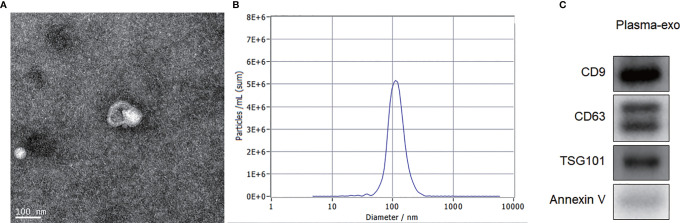
Exosome characterization. **(A)** TEM images showed that the exosomes were clustered and connected with each other, with clear background. The diameter was between 100 nm and 200 nm. The shape was double disc like vesicle structure with intact lipid capsule; **(B)** NTA data revealed that the median value of the total particles was about 100 nm, mainly distributed between 50 and 200nm, and the diameter of a small number of particles was between 0-50 nm; **(C)** Western blotting showed that patient plasma exosomes were positive for the four exosomal markers, Annexin V, Tsg101, CD9 and CD63.

TEM, NTA and western blotting were used to confirm the exosome integrity and purification. The exosomes were obtained by gradient ultracentrifugation at low temperature and then fixed and stained. TEM images showed that the exosomes were clustered and connected with each other, with clear background. The diameter was between 100 and 200 nm. The exosomes had a double disc-like vesicular structure with intact lipid capsule (**Figure 1A**). NTA revealed that the median value of the total particles was about 100 nm, mainly distributed between 50 and 200 nm, and the diameter of a small number of particles was between 0 and 50 nm (**Figure 1B**). Annexin V, TSG101, CD9 and CD63 are common protein markers of exosomes. Western blotting showed that expression of Annexin V, Tsg101, CD9 and CD63 was positive (**Figure 1C**).

### Correlation Between Plasma Exosome-Derived SENP1 Levels and Clinical Pathological Parameters of Melanoma Patients

**Figure 2:**
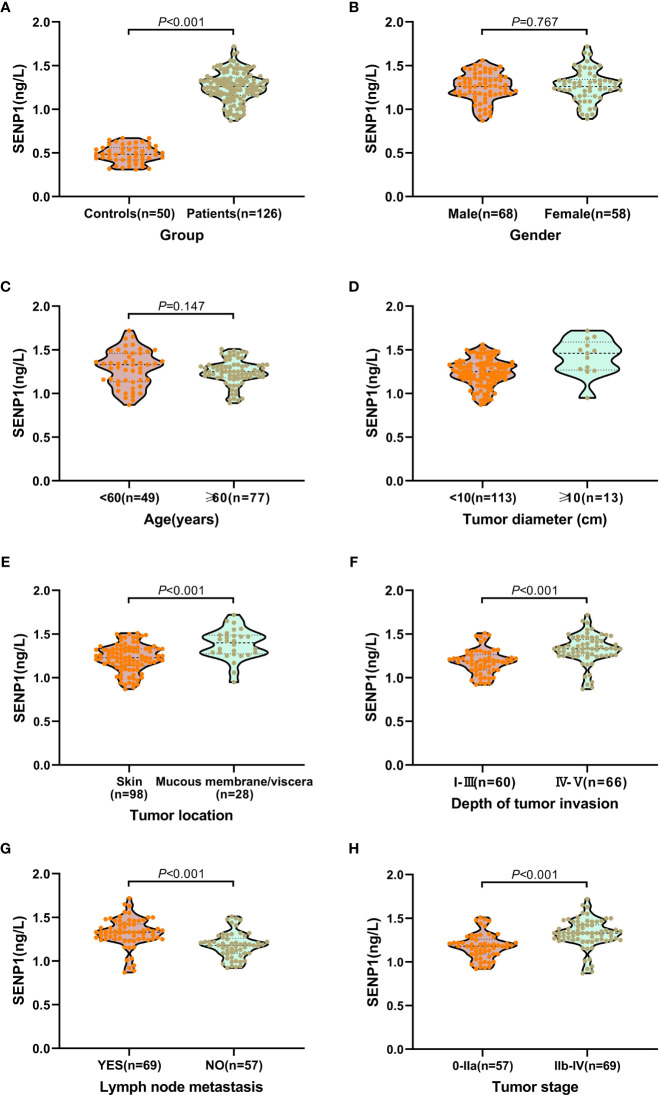
Relationship between plasma exosome-derived SENP1 levels and tumor characteristics. **(A)** The plasma exosome-derived SENP1 levels in melanoma patients were significantly up-regulated than in healthy controls (*P* < 0.001); **(B)** No significant differences were observed between male and female patients (*P* = 0.767). **(C)** No significant differences were found between patients aged < 60 and >60 years (*P* = 0.147). **(D)** The plasma exosome-derived SENP1 levels in melanoma patients with tumor size more than 10 cm were significantly higher than in patients in with tumor size less than 10 cm; **(E)** The plasma exosome-derived SENP1 levels in melanoma patients located in the mucosa or viscera were significantly higher than in patients located in the skin (*P* < 0.001); **(F)** the plasma exosome-derived SENP1 levels in melanoma patients with Clark level IV - V were significantly higher than in patients with Clark level I – III (*P* < 0.001); **(G)** the plasma exosome-derived SENP1 levels in melanoma patients with lymph node metastasis were significantly higher than in patients without lymph node metastasis (*P* < 0.001); **(H)** the plasma exosome-derived SENP1 levels in melanoma patients with TNM stages IIb-IV were significantly higher than in patients with TNM stages IIb-IV (*P* < 0.001).

Plasma exosome-derived SENP1 levels in melanoma patients were significantly upregulated compared with those in healthy controls (*P* < 0.001; **Figure 2A**).

We also investigated the correlations between plasma exosome-derived SENP1 levels and tumor characteristics in melanoma patients. As to sex and age, there was no significant difference in the plasma exosome-derived SENP1 levels between melanoma patients and healthy controls (*P* > 0.05; **Figures 2B, C**). However, plasma exosome-derived SENP1 levels in melanoma patients with tumor size >10 cm, located in the mucosa or viscera, with Clark level IV/V, with lymph node metastasis, and TNM stages IIb–IV were significantly higher than in patients with tumor size <10 cm, located in the skin, with Clark level I–III, without lymph node metastasis, and TNM stages IIb–IV (all *P* < 0.05, **Figures 2D–H**).

**Figure 3:**
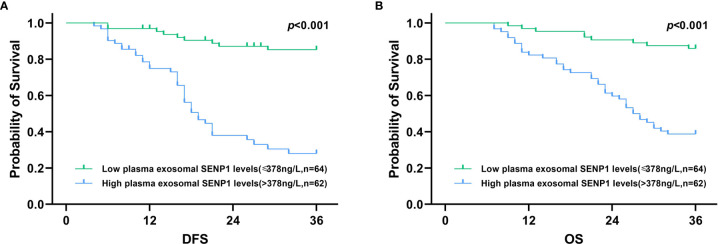
Association of plasma exosome-derived SENP1 levels with DFS and OS in melanoma patients. **(A)** Among all melanoma patients, DFS was worse in melanoma patients who had higher plasma exosome-derived SENP1 levels than those melanoma patients with lower plasma exosome-derived SENP1 levels (*P* < 0.001). **(B)** Among all melanoma patients, OS was worse in melanoma patients who had higher plasma exosome-derived SENP1 levels than those melanoma patients with lower plasma exosome-derived SENP1 levels (*P* < 0.001).

In addition, Kaplan-Meier analysis showed that both DFS and OS were worse in melanoma patients who had higher plasma exosome-derived SENP1 levels than in those with lower plasma exosome-derived SENP1 levels (both *P* < 0.001; **Figure 3**).

### Prognostic Value of Plasma Exosome-Derived SENP1 Levels in Melanoma Patients

We assessed the value of plasma exosome-derived SENP1 for predicting 3-year DFS and OS of melanoma patients with ROC curve analysis (**Table 2**).

**Table 2:** The prognostic value of plasma exosome-derived SENP1 in melanoma patients.

Variable	(n = 126)
**AUROC (3-year DFS)**	**0.82 (0.74-0.88)**
Cutoff value (95%CI)	372
Sensitivity, %	81.2 (69.9-89.6)
Specificity, %	75.4 (62.2-85.9)
Positive predictive value, %	80.0 (68.7-88.6)
Negative predictive value, %	76.8 (63.6-87.0)
Positive likelihood ratio	3.30 (2.1-5.3)
Negative likelihood ratio	0.25 (0.1-0.4)
**AUROC (3-year OS)**	**0.76 (0.67-0.83)**
Cutoff value (95%CI)	366
Sensitivity, %	95.7 (85.5-99.5)
Specificity, %	62.0 (50.4-72.7)
Positive predictive value, %	60.0 (48.0-71.1)
Negative predictive value, %	96.1 (86.5-99.5)
Positive likelihood ratio	2.52 (1.9-3.4)
Negative likelihood ratio	0.069 (0.02-0.3)

**Figure 4:**
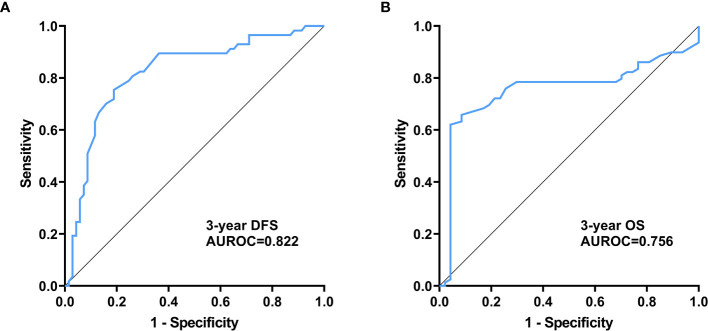
Prognostic value of plasma exosome-derived SENP1 levels in melanoma patients **(A)** AUC for plasma exosome-derived SENP1 levels for predicting 3-year DFS of melanoma patients; **(B)** AUC for plasma exosome-derived SENP1 levels for predicting 3-year OS of melanoma patients.

The area under the ROC curve (AUROC) of plasma exosome-derived SENP1 for predicting 3-year DFS of melanoma patients was 0.82 [95% confidence interval (CI): 0.74–0.88) (**Figure 4A**). With the cutoff value of 372, the positive predictive value and positive likelihood ratio were 80.0 (95% CI: 68.7–88.6) and 3.30 (95% CI: 2.1–5.3), respectively. The negative predictive value and negative likelihood ratio were 76.8 (95% CI: 63.6–87.0) and 0.25 (95% CI: 0.1–0.4), respectively, with a sensitivity of 81.2% (95% CI: 69.9–89.6%) and specificity of 75.4% (95% CI: 62.2–85.9%).

The AUROC of plasma exosome-derived SENP1 for predicting 3-year OS of melanoma patients was 0.76 (95% CI: 0.67–0.83) (**Figure 4B**). With the cut off value of 366, the positive predictive value and positive likelihood ratio were 60.0 (95% CI: 48.0–71.1) and 2.52 (95% CI: 1.9–3.4), respectively. The negative predictive value and negative likelihood ratio were 96.1 (95% CI: 86.5–99.5) and 0.069 (95% CI: 0.02–0.3), respectively, with a sensitivity of 95.7% (95% CI: 85.5–99.5%) and specificity of 62.0% (95% CI: 50.4–72.7%).

## Discussion

Exosomes secreted by malignant tumor cells can carry a variety of characteristic DNA, mRNA, miRNA, long noncoding RNA and proteins into the tumor microenvironment, transfer between tumor cells and normal cells, and stimulate tumor cell proliferation, invasion and metastasis (31). As exosomes contain abundant maternal information and are widely distributed in various body fluids, they are easy to obtain, which is important for early screening, diagnosis and prognostic evaluation of tumors. SENP1 is an important member of the enzyme protein family, which regulates the reverse reaction of SUMO modification.

Previous studies have shown that the overexpression of SENP1 in pancreatic cancer is related to the pathological stage and vascular invasion of patients (21). After gene knockout, the proliferation, migration and invasion of pancreatic cancer cells are inhibited. In gliomas, SENP1 expression is positively correlated with tumor grade. SENP1 is highly expressed in triple-negative breast cancer, which is associated with HER-2 loss (32). In addition, high expression of SENP1 is found in prostate cancer and thyroid oncocytoma. Luo X et al. have showed that miR-541-3p regulates proliferation, migration, and invasion of skin melanoma cell by targeting SENP1 (33). Huang et al. showed that increased SUMOylation of CD45 *via* loss of SENP1 suppresses CD45-mediated dephosphorylation of STAT3, which promotes myeloid-derived suppressor cells development and function, leading to tumorigenesis (34). However, the expression and prognostic value of SENP1 in melanoma patients remain unclear. In addition, it has been proved that exosomes contain many important proteins, which can be used for the early diagnosis of tumor, the prognosis analysis of patients and the targeting of tumor therapy. Hence, we aimed to investigate plasma exosome-derived SENP1 levels and determine their prognostic value in melanoma patients in this study, which is believed to be the first to assess the potential of plasma exosome-derived SENP1 in melanoma diagnosis.

We firstly extracted and characterized exosomes from the plasma of melanoma patients. TEM showed that typical exosomes had oval or bowl-shaped microvesicles. NTA showed that the peak size of plasma exosomes was 50–120 nm. Western blotting showed that the plasma exosomes were positive for Annexin V, Tsg101, CD9 and CD63. We compared the plasma exosome-derived SENP1 levels between melanoma patients and healthy controls. We showed that plasma exosome-derived SENP1 levels in melanoma patients were significantly upregulated, which is consistent with the trend in SENP1 protein expression in melanoma patients.

We also investigated the correlations between plasma exosome-derived SENP1 levels and tumor characteristics in melanoma patients. The plasma exosome-derived SENP1 levels were related to tumor size, tumor location, depth of tumor invasion, lymph node metastasis, and TNM stage. The plasma exosome-derived SENP1 levels in melanoma patients with tumor size >10 cm, located in the mucosa or viscera, with Clark level IV/V, lymph node metastasis, and TNM stages IIb–IV were significantly higher than in patients in with tumor size <10 cm, located in the skin, with Clark level I–III, without lymph node metastasis, and TNM stages IIb–IV. In addition, both DFS and OS were worse in melanoma patients who had higher plasma exosome-derived SENP1 levels than in those with lower plasma exosome-derived SENP1 levels. We showed that plasma exosome-derived SENP1 had good performance for predicting 3-year DFS and 3-year OS of melanoma patients.

There were some limitations to this study. First, although this was a large study to evaluate the plasma exosome-derived SENP1 levels in melanoma patients, its prognostic value needs to be verified by multicenter and larger samples. Second, the mechanism of plasma exosome-derived SENP1 affecting the prognosis of melanoma patients needs to be studied *in vivo* and *in vitro*. Third, melanoma patients with different treatment regimens would have an impact on the prognosis. We need to expand the number of cases and group them according to different treatment regimens, so as to eliminate the impact of treatment regimens on the results. And also, we determined the expression of SENP1 in the tissues of melanoma patients by IHC, and found that the SENP1 protein level was significantly higher than that in the adjacent tissues, but its relationship with the prognosis of patients was not obvious.

In summary, our study demonstrated that melanoma patients with higher plasma exosome-derived SENP1 levels had worse DFS and OS. The plasma exosome-derived SENP1 levels may be a potential prognostic predictor for 3-year DFS and OS of melanoma patients.

## Data Availability Statement 

The original contributions presented in the study are included in the article/supplementary material. Further inquiries can be directed to the corresponding author.

## Author Contributions 

HH, BL, YS, and HW contributed to study concept and design, acquisition of data, analysis and interpretation of data, and drafting of the manuscript. BZ and YM contributed to statistical analysis. GZ contributed to study concept and design, study supervision and critical revision of the manuscript. All authors contributed to the article and approved the submitted version.

## Funding

This work was supported by the outstanding young backbone teachers project of “Qinglan Project” in Jiangsu Province (No. 2018) and the research project fund of the Institute level of Suzhou Vocational Health College (No: szwzy201708).

## Conflict of Interest

The authors declare that the research was conducted in the absence of any commercial or financial relationships that could be construed as a potential conflict of interest.

## Publisher’s Note

All claims expressed in this article are solely those of the authors and do not necessarily represent those of their affiliated organizations, or those of the publisher, the editors and the reviewers. Any product that may be evaluated in this article, or claim that may be made by its manufacturer, is not guaranteed or endorsed by the publisher.

## Abbreviations

SENP1, Sentrin sumo-specific protease 1; SUMO, Small ubiquitin-related modifier; NTA, Nanoparticle tracking analysis; TEM, Transmission electron microscopy; DFS, Disease-free survival; OS, Overall survival.

## References

[1] Armanetti P Chillà A Margheri F Biagioni A Menichetti L Margheri G. Enhanced Antitumoral Activity and Photoacoustic Imaging Properties of AuNP-Enriched Endothelial Colony Forming Cells on Melanoma. Adv Sci (Weinh) (2021) 8:2001175. 10.1002/advs.202001175 33643785 PMC7887578

[2] Dong X Song J Chen B Qi Y Jiang W Li H. Exploration of the Prognostic and Immunotherapeutic Value of B and T Lymphocyte Attenuator in Skin Cutaneous Melanoma. Front Oncol (2020) 10:592811. 10.3389/fonc.2020.592811 33718105 PMC7953043

[3] Mojtahed Saam A Boyer Nicole R Rao Saieesh A Gajewski TF Tseng J Turaga KK. Cost-Effectiveness Analysis of Adjuvant Therapy for BRAF-Mutant Resected Stage III Melanoma in Medicare Patients. Ann Surg Oncol (2021). 10.1245/s10434-021-10288-4 34129153

[4] Marusak C Thakur V Li Y Freitas JT Zmina PM Thakur VS. Targeting Extracellular Matrix Remodeling Restores BRAF Inhibitor Sensitivity in BRAFi-Resistant Melanoma. Clin Cancer Res (2020) 26:6039–50. 10.1158/1078-0432.CCR-19-2773 PMC7669662 32820016

[5] Odiase O Noah-Vermillion L Simone BA Aridgides PD. The Incorporation of Immunotherapy and Targeted Therapy Into Chemoradiation for Cervical Cancer: A Focused Review. Front Oncol (2021) 11:663749. 10.3389/fonc.2021.663749 34123823 PMC8189418

[6] Ohkuma K Yoshino K Morita H Sugimoto E Hiura A Uehara J. Pigmented Circumscribed Plantar Hypokeratosis Mimicking Acral Lentiginous Melanoma: A Case Report. J Dermatol (2021) 48:e108–9. 10.1111/1346-8138.15715 33617012

[7] Keilholz U Ascierto PA Dummer R Robert C Lorigan P van Akkooi A. ESMO Consensus Conference Recommendations on the Management of Metastatic Melanoma: Under the Auspices of the ESMO Guidelines Committee. Ann Oncol (2020) 31:1435–48. 10.1016/j.annonc.2020.07.004 32763453

[8] Robert C Hwu W-J Hamid O Ribas A Weber JS Daud AI. Long-Term Safety of Pembrolizumab Monotherapy and Relationship With Clinical Outcome: A Landmark Analysis in Patients With Advanced Melanoma. Eur J Cancer (2021) 144:182–91. 10.1016/j.ejca.2020.11.010 PMC8388128 33360855

[9] Ronchi A Pagliuca F Zito Marino F Argenziano G Brancaccio G Alfano R. Second Diagnostic Opinion by Experienced Dermatopathologists in the Setting of a Referral Regional Melanoma Unit Significantly Improves the Clinical Management of Patients With Cutaneous Melanoma. Front Med (Lausanne) (2020) 7:568946. 10.3389/fmed.2020.568946 33614670 PMC7890120

[10] Pitney T Muir Dr J. Single-Center, Single-Operator, Retrospective Analysis of Base Transection Rates in Shave Procedures for Melanoma Diagnosis. J Am Acad Dermatol (2021) 84:861–2. 10.1016/j.jaad.2020.10.049 33616070

[11] Barham W Guo R Park Sean S Herrmann J Dong H Yan Y. Case Report: Simultaneous Hyperprogression and Fulminant Myocarditis in a Patient With Advanced Melanoma Following Treatment With Immune Checkpoint Inhibitor Therapy. Front Immunol (2020) 11:561083. 10.3389/fimmu.2020.561083 33603731 PMC7884751

[12] Cheng S Li Z Zhang W Sun Z Fan Z Luo J. Identification of IL10RA by Weighted Correlation Network Analysis and *In Vitro* Validation of Its Association With Prognosis of Metastatic Melanoma. Front Cell Dev Biol (2020) 8:630790. 10.3389/fcell.2020.630790 33490091 PMC7820192

[13] JiXu A Dinnes J Matin RN. Total Body Photography for the Diagnosis of Cutaneous Melanoma in Adults: A Systematic Review and Meta-Analysis. Br J Dermatol (2020). 10.1111/bjd.19759 33369727

[14] Le X Negrao Marcelo V Reuben A Federico L Diao L McGrail D. Characterization of the Immune Landscape of EGFR-Mutant NSCLC Identifies CD73/adenosine Pathway as a Potential Therapeutic Target. J Thorac Oncol (2020) 16:583–600. 10.1016/j.jtho.2020.12.010 33388477 PMC11160459

[15] van Poppelen Natasha M de Bruyn Daniël P Bicer T Verdijk R Naus N Mensink H. Genetics of Ocular Melanoma: Insights Into Genetics, Inheritance and Testing. Int J Mol Sci (2020) 22:336. 10.3390/ijms22010336 PMC7795687 33396957

[16] Kim Y-R Jacobs Julia S Li Q Gaddam RR Vikram A Liu J. SUMO2 Regulates Vascular Endothelial Function and Oxidative Stress in Mice. Am J Physiol Heart Circ Physiol (2019) 317:H1292–300. 10.1152/ajpheart.00530.2019 PMC8906825 31584834

[17] Chen S Dong D Xin W Zhou H. Progress in the Discovery of Small Molecule Modulators of DeSUMOylation. Curr Issues Mol Biol (2020) 35:17–34. 10.21775/cimb.035.017 31422931

[18] Namuduri Arvind V Heras G Lauschke VM Vitadello M Traini L Cacciani N. Expression of SUMO Enzymes is Fiber Type Dependent in Skeletal Muscles and Is Dysregulated in Muscle Disuse. FASEB J (2020) 34:2269–86. 10.1096/fj.201901913R 31908008

[19] Filippopoulou C Simos G Chachami G. The Role of Sumoylation in the Response to Hypoxia: An Overview. Cells (2020) 9:2359. 10.3390/cells9112359 PMC7693722 33114748

[20] Hotz W Wiesnet M Tascher G Braun T Müller S Mendler L. Profiling the Murine SUMO Proteome in Response to Cardiac Ischemia and Reperfusion Injury. Molecules (2020) 25:5571. 10.3390/molecules25235571 PMC7731038 33260959

[21] Bouchard Danielle M Matunis Michael J. A Cellular and Bioinformatics Analysis of the SENP1 SUMO Isopeptidase in Pancreatic Cancer. J Gastrointest Oncol (2019) 10:821–30. 10.21037/jgo.2019.05.09 PMC6776818 31602319

[22] Heo K-S. Regulation of Post-Translational Modification in Breast Cancer Treatment. BMB Rep (2019) 52:113–8. 10.5483/BMBRep.2019.52.2.017 PMC6443327 30638182

[23] Ambaye Nigus D. Noncovalent Structure of SENP1 in Complex With SUMO2. Acta Crystallogr F Struct Biol Commun (2019) 75:332–9. 10.1107/S2053230X19004266 PMC6497105 31045562

[24] Zhou M Bian Z Liu B Zhang Y Cao Y Cui K. Long Noncoding RNA MCM3AP-AS1 Enhances Cell Proliferation and Metastasis in Colorectal Cancer by Regulating miR-193a-5p/SENP1. Cancer Med (2021) 10:2470–81. 10.1002/cam4.3830 PMC7982620 33686713

[25] Wang L Wu J Song S Chen H Hu Y Xu B. Plasma Exosome-Derived Sentrin SUMO-Specific Protease 1: A Prognostic Biomarker in Patients With Osteosarcoma. Front Oncol (2021) 11:625109. 10.3389/fonc.2021.625109 33791211 PMC8006461

[26] Catalano M O’Driscoll L. Inhibiting Extracellular Vesicles Formation and Release: A Review of EV Inhibitors. J Extracell Vesicles (2020) 9:1703244. 10.1080/20013078.2019.1703244 32002167 PMC6968539

[27] Chen K Wang Q Kornmann M Tian X Yang Y. The Role of Exosomes in Pancreatic Cancer From Bench to Clinical Application: An Updated Review. Front Oncol (2021) 11:644358. 10.3389/fonc.2021.644358 33718244 PMC7952979

[28] Du Q Ye X Lu S-R Li H Liu HY Zhai Q. Exosomal miR-30a and miR-222 Derived From Colon Cancer Mesenchymal Stem Cells Promote the Tumorigenicity of Colon Cancer Through Targeting MIA3. J Gastrointest Oncol (2021) 12:52–68. 10.21037/jgo-20-513 33708424 PMC7944155

[29] Müller Bark J Kulasinghe A Amenábar JM Punyadeera C. Exosomes in Cancer. Adv Clin Chem (2021) 101:1–40. 10.1016/bs.acc.2020.06.006 33706886

[30] Mondal Sujan K Whiteside Theresa L. Immunoaffinity-Based Isolation of Melanoma Cell-Derived and T Cell-Derived Exosomes From Plasma of Melanoma Patients. Methods Mol Biol (2021) 2265:305–21. 10.1007/978-1-0716-1205-7_23 PMC9113061 33704724

[31] Wu J Chen Z-P Shang A-Q Wang WW Chen ZN Tao YJ. Systemic Bioinformatics Analysis of Recurrent Aphthous Stomatitis Gene Expression Profiles.[J]. Oncotarget (2017) 8:111064–72. 10.18632/oncotarget.22347 PMC5762305 29340037

[32] Wang Z Jin J Zhang J Wang L Cao J. Depletion of SENP1 Suppresses the Proliferation and Invasion of Triple-Negative Breast Cancer Cells. Oncol Rep (2016) 36:2071–8. 10.3892/or.2016.5036 27573572

[33] Luo X Chen JY. The Mechanism of miR-541-3p Regulates Proliferation, Migration, and Invasion of Skin Melanoma Cell by Targeting SENP1. J Guangxi Med Univ (2020) 37(7):1201–8.

[34] Huang X Zuo Y Wang X Wu X Tan H Fan Q. SUMO-Specific Protease 1 Is Critical for Myeloid-Derived Suppressor Cell Development and Function. Cancer Res (2019) 79:3891–902. 10.1158/0008-5472.CAN-18-3497 31186231

